# Veterinary deworming agent-induced toxic optic neuropathy: a case report

**DOI:** 10.1186/s12886-023-03097-8

**Published:** 2023-08-16

**Authors:** Lin Qin, Kan Wang, Yang Ou, Long Pang

**Affiliations:** 1 Department of Ophthalmology, Guangdong Provincial Hospital of Chinese Medicine, Guangzhou, 510000 China; 2 The Second Clinical College, Guangzhou University of Chinese Medicine, Guangzhou, 510120 Guangdong China

**Keywords:** Bithionol, Case report, Ocular, Optic neurotoxicity, Toxic optic neuropathy, Triclabendazole, Vision

## Abstract

**Background:**

Veterinary antiparasitic drugs are widely used in countries and regions in which parasitic diseases are endemic, which leads to the risk of accidental ingestion and poisoning in humans.

**Case presentation:**

A 40-year-old male patient with a history of cirrhosis sought medical attention on November 25, 2021, due to progressive vision loss. He had previously taken triclabendazole and bithionol and was diagnosed with toxic optic neuropathy on examination. Steroid, neurotonic, and high-pressure oxygen therapy were ineffective.

**Conclusions:**

Triclabendazole and bithionol have potential risk of optic neurotoxicity and should be considered for enhanced supervision and warning labels.

## Background

Endemic parasitic diseases present a major public health problem, which still affect developing countries. Even though some old antiparasitic drugs such as triclabendazole, bithionol, and niclofolan are no longer, or rarely, used in humans, a many of these drugs are used to treat parasitic diseases in livestock. Veterinary antiparasitic drugs are widely available in countries and regions with endemic parasitic diseases and, with the development of e-commerce, these drugs are easily purchased on the Internet. There have been many reports of accidental ingestion of antiparasitic drugs in humans, resulting in poisoning. This includes optic neurotoxicity caused by closantel and niclofolan [1–3], however there have been no reports of optic neurotoxicity caused by triclabendazole and bithionol.

## Case presentation

**Fig. 1:**
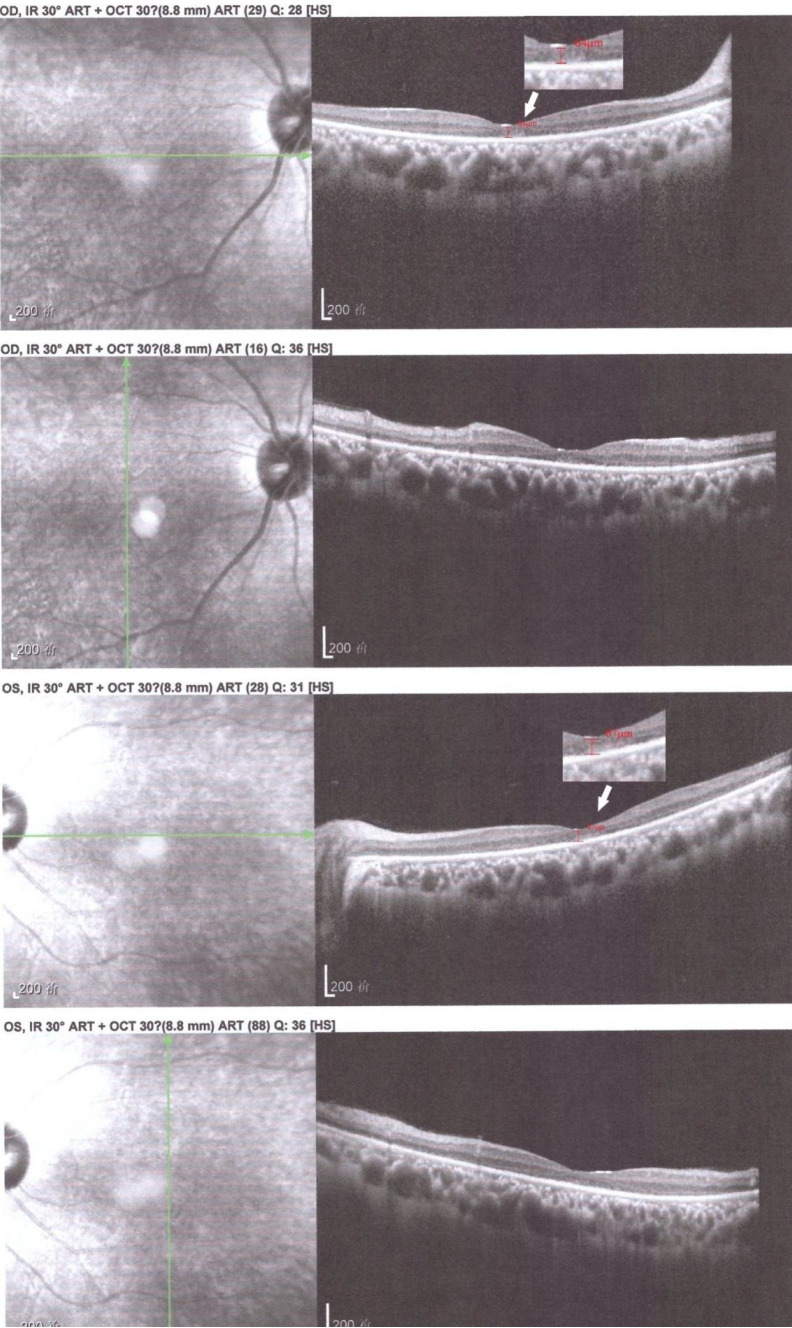
OCT inspection conducted on November 26th, 2021, about 3 weeks after taking triclabendazole and bithionol. OCT showed bilateral macular retinal thinning(OD:64 μm; OS:67 μm), and disappearance of the outer retinal reflex

**Fig. 2:**
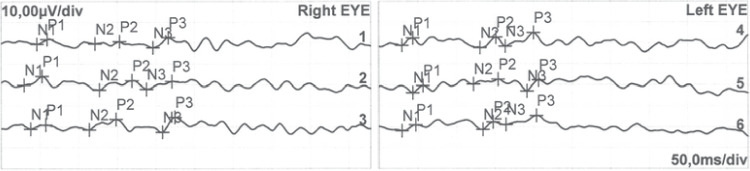
FVEP conducted on November 26th, 2021, about 3 weeks after taking triclabendazole and bithionol. FVEP showed a delay and decrease in amplitude of the P wave peak

**Fig. 3:**
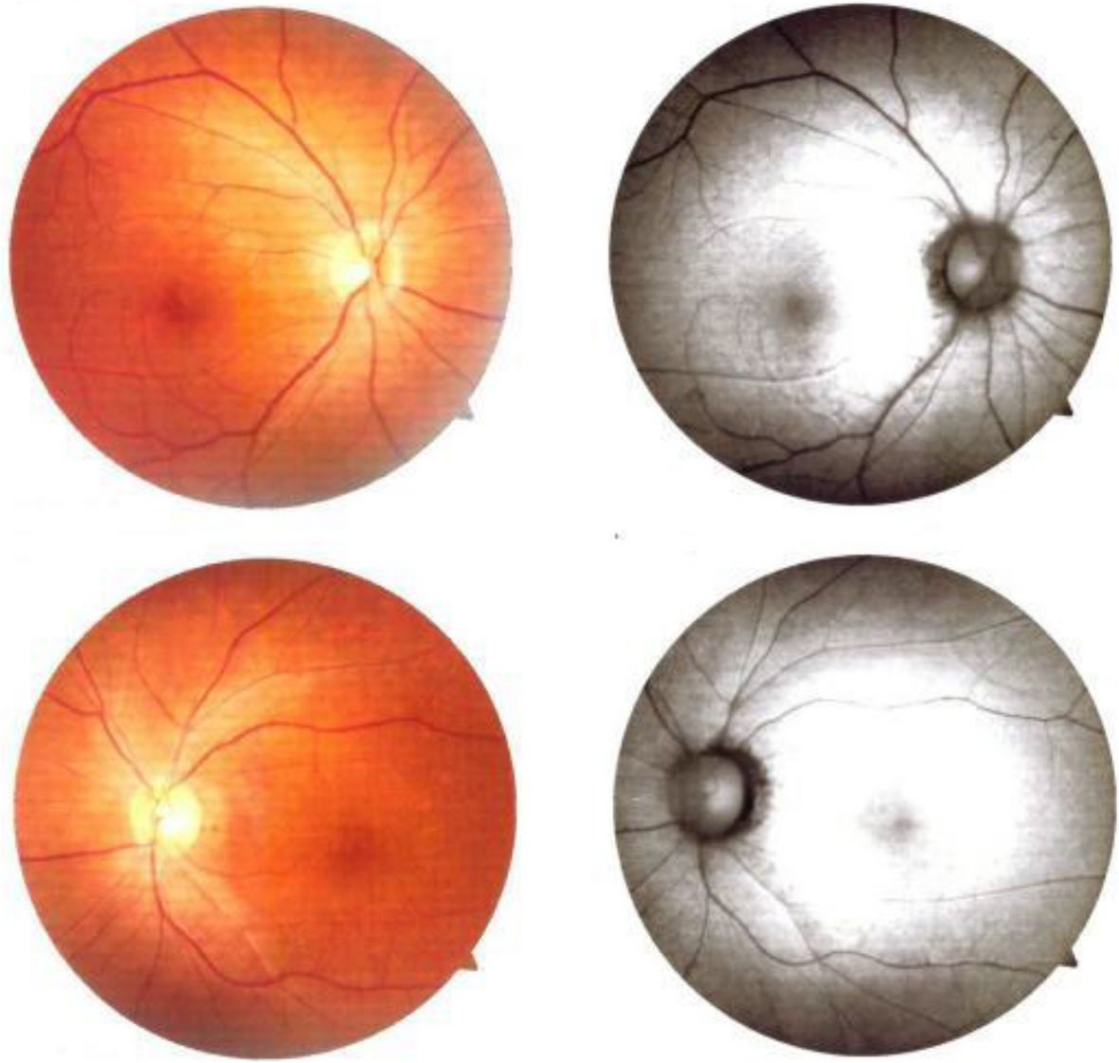
Fundus color photography conducted on November 26th, 2021, about 3 weeks after taking triclabendazole and bithionol. Fundus color photography showed both optic discs appeared pale

A 40-year-old male patient sought medical attention at our hospital on November 25, 2021, due to progressive vision loss. According to his medical history, the patient suspected that he had parasitic worms and took the veterinary antiparasitic drug triclabendazole 0.7 g and bithionol 1.5 g, which he had purchased online, around 20 days ago. After taking the drug, he drank 500 ml of beer. After 2 days, he developed mild blurred vision in both eyes. After 1 week, he felt that the bilateral blurred vision had worsened. After 2 weeks, he experienced bilateral visual impairment accompanied by micturition and bowel movement difficulties and sought medical attention at a local hospital. No apparent abnormalities were observed in cranial and bilateral orbital MRI with contrast. Visual acuity did not improve after steroid and neurotonic treatments(dexamethasone 10 mg iv qd, mecobalamin 0.5 mg tid po), thus he sought medical attention at our hospital. The patient had a multi-year history of hepatitis B, cirrhosis, and gastric fundal varices. In October 2020, he was hospitalized for treatment due to liver failure and was on long-term entecavir, glutathione, polyene phosphatidyl choline, and ursodiol treatment. His hepatic function was stable and he was not taking other drugs. On admission, the patient was conscious and had bilateral visual impairment accompanied by micturition and bowel movement difficulties. Physical examination showed that light perception was absent in both eyes. Intraocular pressure was 12.0 mmHg and 13.7 mmHg in the right and left eyes, respectively. Bilateral pupil diameter was 4.5 mm, light reflex was absent, the optic disc boundary was clear, the fundus was pale-red in color, the C/D was around 0.3–0.4, and the retina was flat. There was no fundus bleeding or exudation and the fovea was reflective. Macular OCT showed bilateral macular retinal thinning, and disappearance of the outer retinal reflex (Fig. 1). Optic nerve OCT examination showed that RNFL thickness on the temporal side of the right eye was slightly thinner, as well as the nasal side of the left eye. However, due to the patient’s poor visual acuity and poor fixation during OCT examination, the above data did not pass baseline verification. Bilateral ocular FVEP showed a delay and decrease in amplitude of the P wave peak (Fig. 2). Fundus color photography showed both optic discs appeared pale (Fig. 3). The neurotonics citicoline (0.1 g bid po) and idebenone (30 mg tid po) were given and high-pressure oxygen therapy was administered. After 1 week of treatment, the patient’s bilateral visual acuity did not improve, and he was discharged. The patient’s visual acuity still had not improved after half a year of follow-up.

## Discussion and conclusion

For treating fascioliasis and paragonimiasis in humans, the WHO recommends a dose of 10 mg/kg oral triclabendazole and the FDA recommends a total dose of 20 mg/kg, taken in two doses [4]. Adverse reactions to triclabendazole mainly include abdominal pain, biliary colic, transaminase elevation, and urticaria [4]. Before triclabendazole, bithionol was used as the first line antiparasitic drug. However, bithionol was discontinued due to its long treatment course, poor adherence, many side effects, and unclear therapeutic effects [5]. According to previous reports, the oral dose of bithionol is 25–50 mg/(kg·d) and 10–15 doses are taken. The incidence of side effects with bithionol is 41.2% and include mainly abdominal pain, diarrhea, and rashes [6]. Serious adverse reactions to bithionol have not been reported. One study reported improvements in visual acuity and visual field after bithionol treatment in patients with acute phases of cerebral paragonimiasis [7], indicating that the drug does not cause significant optic nerve toxicity in patients with normal hepatic and renal function. The patient in the current study took doses of oral triclabendazole and bithionol for humans with normal hepatic and renal function (11.9 mg/kg and 25.4 mg/kg, respectively), but developed progressive and irreversible vision loss. Since retinal hemorrhage, exudation, and optic disc edema, were not seen upon fundoscopy and optic nerve changes were not found MRI of the cranium and orbital, drug-induced toxic optic neuropathy was considered. Even though serious adverse reactions to triclabendazole and bithionol have not been reported clinically thus far [4, 8, 9], there have been no studies of the use of these drugs in patients with hepatic and renal insufficiency [5]. Since triclabendazole and bithionol are metabolized by the liver [10], the synergistic effects of underlying cirrhosis and alcohol use may have increased the toxicity of these two drugs. In addition, drug-drug interactions between triclabendazole and bithionol, as well as between antiparasitic drugs and anti-hepatitis B and hepatoprotective drugs are unclear. Further study regarding adverse interactions between these drugs is required to determine whether toxicity due to drug interactions. Steroids, neurotonic drugs, and early renal replacement treatment have been reported to be effective in treating closantel-induced optic neurotoxicity [2, 11–13]; however, vision loss did not improve in our patient after steroid and neurotonic treatment. The half-lives of triclabendazole and its active metabolites, sulfoxide, and sulfone, are 8, 14, and 11 h, respectively [10]. The concentration of bithionol in blood is significantly lower than in bile in which it reaches peak concentration 2 h after administration [14]. CRRT was not performed in our patient as more than 20 days had passed when he sought medical attention at our hospital.

In summary, this case study indicates that there is a potential risk for optic neurotoxicity with the use of triclabendazole and bithionol. Patients with inadequate liver function cannot use these drugs without the approval of a doctor, and patients taking these drugs should be closely monitored for possible optic neurotoxicity These drugs should not be allowed to be sold on the internet.

## Data Availability

The datasets used and/or analysed during the current study are available from the corresponding author on reasonable request.

## Abbreviations

MRI: Magnetic resonance imaging
OCT: Optical Coherence Tomography
RNFL: Retinal nerve fiber layer
FVEP: Flash visual evoked potential
WHO: World Health Organization
FDA: Food and Drug Administration
CRRT: Continuous renal replacement therapy
